# Supplementary material for: Reputation effects drive the joint evolution of cooperation and social rewarding
Source: Nat Commun. 2022 Oct 7;13:5928. doi: 10.1038/s41467-022-33551-y (PMC9547006; doi:10.1038/s41467-022-33551-y)
Supplement: Supplementary file 1 — Supplementary Information [file 41467_2022_33551_MOESM1_ESM.pdf]

Supplementary Information

Reputation effects drive the joint evolution of cooperation and social rewarding

Saptarshi Pal[1] and Christian Hilbe[1]

[1]Max Planck Research Group Dynamics of Social Behavior, Max Planck Institute for Evolutionary Biology, 24306 Ploen, Germany

In the following, we characterize the Nash equilibria of the two-player and multi-player game. In a Nash equilibrium, no player has an incentive to deviate. That is, no player can gain a higher payoff by unilaterally switching to a different strategy, given the other players' strategies.

1 Supplementary Note 1: Equilibrium analysis of the two-player game

1.1 Description of the game

As in the main text, we consider a game with two stages and with two players, a donor and a recipient. In the first stage, the donor decides whether to cooperate or to defect. Cooperation means to pay a cost $c > 0$ to transfer a benefit $b > 0$ to the recipient. In the second stage, the recipient decides whether or not to reward the donor. For the baseline model, we assume that rewarding is costly; that is, a rewarding recipient pays a cost $\gamma > 0$ in order to transfer a benefit $\beta > 0$ to the donor. The recipient's decision may be contingent on the donor's action in the first stage. As a result, the recipient has four strategies:

(i) Never rewarding (reward no one; *NR*),

(ii) Social rewarding (only reward cooperators; *SR*),

(iii) Antisocial rewarding (only reward defectors *AR*),

(iv) Unconditional rewarding (reward everyone; *UR*).

For the first stage, we assume donors know a recipient's strategy with some exogenous probability λ. We refer to λ as the population's *information transmissibility*. When donors know the recipient's strategy, they can act opportunistically. Specifically, they may only cooperate against those recipients who insist on cooperation to offer a reward. Overall, this assumption gives rise to four natural donor strategies:

(i) Unconditional cooperation (*C*),

(ii) Opportunistic cooperation (if the recipient's strategy is unknown, the donor cooperates; otherwise the donor cooperates if and only if the recipient is known to reward socially; *OC*),

(iii) Opportunistic defection (if the recipient's strategy is unknown, the donor defects; otherwise the donor cooperates if and only if the recipient is known to reward socially; *OD*),

(iv) Unconditional defection (*D*).

We note that in general, there are additional strategies for donors; for example, donors may cooperate if and only if the recipient is known to reward antisocially. However, it is easy to see that such strategies are (weakly) dominated. Hence, we will not consider them here.

The resulting interaction between donors and recipients can be represented as a bimatrix game. The first matrix A represents the payoff of each possible donor strategy when interacting against a given recipient strategy,

$$\begin{array}{c} \\ C \\ OC \\ OD \\ D \end{array} \begin{array}{c} \begin{array}{cccc} NR & SR & AR & UR \end{array} \\ \begin{pmatrix} -c & \beta - c & -c & \beta - c \\ -\bar{\lambda}c & \beta - c & \lambda\beta - \bar{\lambda}c & \beta - \bar{\lambda}c \\ 0 & \lambda(\beta - c) & \beta & \beta \\ 0 & 0 & \beta & \beta \end{pmatrix} \end{array}. \tag{1}$$

Here, $\bar{\lambda} := 1 - \lambda$ is the shortcut notation for the probability that a donor does not know the recipient's strategy ahead of the game. Similarly, the second matrix B represents the payoffs of the recipient,

$$\begin{array}{c} \\ C \\ OC \\ OD \\ D \end{array} \begin{array}{c} \begin{array}{cccc} NR & SR & AR & UR \end{array} \\ \begin{pmatrix} b & b - \gamma & b & b - \gamma \\ \bar{\lambda}b & b - \gamma & \bar{\lambda}b - \lambda\gamma & \bar{\lambda}b - \gamma \\ 0 & \lambda(b - \gamma) & -\gamma & -\gamma \\ 0 & 0 & -\gamma & -\gamma \end{pmatrix} \end{array}. \tag{2}$$

To describe the Nash equilibria of this bimatrix game, it is useful to allow players to use mixed strategies. For the donor, mixed strategies take the form $\mathbf{x} = (x_C, x_{OC}, x_{OD}, x_D)$; here, x_i is the probability that the donor uses the pure strategy i against a given recipient. Similarly, we denote a recipient's mixed strategy by $\mathbf{y} = (y_{NR}, y_{SR}, y_{AR}, y_{UR})$. As usual, the entries of $\mathbf{x}$ and $\mathbf{y}$ need to add up to one. Using this notation, we can define the payoff $\pi_{\mathbf{DO}}$ of the donor and the payoff $\pi_{\mathbf{RE}}$ of the recipient as follows[1],

$$\begin{aligned} \pi_{\mathbf{DO}}(\mathbf{x}, \mathbf{y}) &= \mathbf{x}\, A\, \mathbf{y}^\top, \\ \pi_{\mathbf{RE}}(\mathbf{x}, \mathbf{y}) &= \mathbf{x}\, B\, \mathbf{y}^\top. \end{aligned} \tag{3}$$

We associate pure strategies with the respective vectors $\mathbf{x}$ and $\mathbf{y}$ that place full weight on the respective pure strategy; as an example, we associate the donor's strategy *OC* with the vector $\mathbf{x} = (0, 1, 0, 0)$.

In the following, we describe all equilibria of this bimatrix game (in which the player's roles are

fixed). We note that the corresponding equilibria are naturally related to the equilibria of the symmetrized game, in which players can take either role with equal probability (which we use for our evolutionary simulations). For example, the strategy profile (*OC*, *SR*) is a Nash equilibrium of the asymmetric game if and only if the strategy (*OC*, *SR*) is a Nash equilibrium of the symmetrized game. In the first case, the interpretation of (*OC*, *SR*) is that the donor uses the strategy *OC* and recipient uses strategy *SR*; in the second case, the interpretation of (*OC*, *SR*) is that a player uses strategy *OC* when in the role of the donor and strategy *SR* when in the role of the recipient. For more details on the relationship between asymmetric games and their symmetrized counterparts, see Section 2.5 of the book by Sigmund[1].

The following result shows that the interaction between donors and receivers is only interesting when both cooperation and rewards are sufficiently profitable.

Proposition 1. *If either the cost of cooperation is not compensated by the reward ($\beta < c$), or if the cost of rewarding is not compensated by the benefit of cooperation ($b < \gamma$), there is no Nash equilibrium in which donors cooperate, or in which recipients reward.*

We provide all proofs in the Supplementary Note 3. The result is intuitive: if rewards are too small, there is no reason to be swayed by the prospects of being rewarded. On the other hand, when the benefit of cooperation is too small, there is no incentive to provide excessive rewards. Based on Proposition 1, in the following we restrict ourselves to cases in which both cooperation and rewards are sufficiently profitable to incentivize social behavior. That is, we assume $b > \gamma$ and $\beta > c$ throughout.

1.2 Characterization of Nash equilibria

Before characterizing the Nash equilibria directly, we first prove the following auxiliary result that is useful to reduce the number of cases to consider.

Lemma 1. *Suppose the parameters satisfy $0 < c < b$ and $0 < \gamma < \beta$.*

1. *For all strategies $\mathbf{y}$ of the recipient, the donor's payoffs satisfy*

$$\pi_{\mathbf{DO}}(\mathrm{C}, \mathbf{y}) \leq \pi_{\mathbf{DO}}(\mathrm{OC}, \mathbf{y}) \quad \textit{and} \quad \pi_{\mathbf{DO}}(\mathrm{D}, \mathbf{y}) \leq \pi_{\mathbf{DO}}(\mathrm{OD}, \mathbf{y}). \tag{4}$$

   The first inequality is strict if and only if $\lambda > 0$ and $y_{\mathrm{SR}} < 1$. The second inequality is strict if and only if $\lambda > 0$ and $y_{\mathrm{SR}} > 0$.

2. *For all mixed strategies $\mathbf{x}$ of the donor, the recipient's payoffs satisfy*

$$\pi_{\mathbf{RE}}(\mathbf{x}, \mathrm{AR}) \leq \pi_{\mathbf{RE}}(\mathbf{x}, \mathrm{NR}) \quad \textit{and} \quad \pi_{\mathbf{RE}}(\mathbf{x}, \mathrm{UR}) < \pi_{\mathbf{RE}}(\mathbf{x}, \mathrm{NR}). \tag{5}$$

   The first inequality is strict if and only if $x_{\mathrm{C}} = 1$ (when $\lambda > 0$) or $x_{\mathrm{C}} + x_{\mathrm{OC}} = 1$ (when $\lambda = 0$).

The first part of Lemma 1 states that for the donor, all unconditional strategies are weakly dominated by their respective opportunistic counterparts. The result is again intuitive – if there is reliable information about the recipient's future behavior, it is optimal to take this information into account, and to adapt one's strategy accordingly. The second part states that for the recipient, antisocial rewarding is weakly dominated, whereas unconditional rewarding is strictly dominated. Also this result is intuitively clear – since rewarding is costly, it only pays if it makes the donor more likely to cooperate. However, among all the recipient's strategies, only social rewarding has this positive influence, but not antisocial or unconditional rewarding.
Using the above result, we can characterize the pure Nash equilibria of the game as follows.

Proposition 2. *Suppose the parameters satisfy* $0 < c < b$ *and* $0 < \gamma < \beta$*, and let* $(\mathbf{x}, \mathbf{y})$ *be a strategy profile. Then* $(\mathbf{x}, \mathbf{y})$ *is a pure Nash equilibrium if and only if one of the following four cases applies:*

$$(i)\ (\mathbf{x}, \mathbf{y}) = (\mathrm{D}, \mathrm{NR}) \qquad (ii)\ (\mathbf{x}, \mathbf{y}) = (\mathrm{OC}, \mathrm{SR})\ \textit{and}\ \lambda \geq \gamma/b$$
$$(iii)\ (\mathbf{x}, \mathbf{y}) = (\mathrm{OD}, \mathrm{SR})\ \textit{and}\ \lambda = 1, \qquad (iv)\ (\mathbf{x}, \mathbf{y}) = (\mathrm{OD}, \mathrm{NR})\ \textit{and}\ \lambda = 0.$$

In particular, Proposition 2 implies that for intermediate information transmissibilities ($0 < \lambda < 1$) there is either only one pure Nash equilibrium (*D*, *NR*), or there are two Nash equilibria – with (*OC*, *SR*) being the second Nash equilibrium. The first case arises when donors are comparably unlikely to be informed of the recipient's strategy (i.e., when $\lambda < \gamma/b$). The second case arises when this inequality is reversed. The remaining two cases are non-generic, as they require specific values of λ to arise. Moreover, these two cases are qualitatively similar to the first two cases, in the sense that case (*iii*) leads to the same behavior as case (*ii*), and case (*iv*) brings about the same behavior as case (*i*).

Similarly, we can also characterize all mixed Nash equilibria.

Proposition 3. *Suppose the parameters satisfy* $0 < c < b$ *and* $0 < \gamma < \beta$*, and let* $(\mathbf{x}, \mathbf{y})$ *be a strategy profile. Then* $(\mathbf{x}, \mathbf{y})$ *is a mixed Nash equilibrium if and only if one of the following cases applies:*

(i) $x_{\mathrm{C}} + x_{\mathrm{OC}} = 1$ *and* $y_{\mathrm{SR}} = 1$, *where* $x_{\mathrm{C}} \leq 1 - \frac{\gamma}{\lambda b}$ *and* $\lambda \geq \frac{\gamma}{b}$.

(ii) $x_{\mathrm{OC}} = \frac{\lambda(b-\gamma)}{(1-\lambda)\gamma}$, $x_{\mathrm{OD}} = 1 - \frac{\lambda(b-\gamma)}{(1-\lambda)\gamma}$ *and* $y_{\mathrm{NR}} = 1 - \frac{c}{\beta}$, $y_{\mathrm{SR}} = \frac{c}{\beta}$ *for* $\lambda \leq \frac{\gamma}{b}$.

(iii) $x_{\mathrm{OD}} + x_{\mathrm{D}} = 1$ *and* $y_{\mathrm{NR}} + y_{\mathrm{SR}} = 1$, *where* $y_{\mathrm{NR}} \geq 1 - \frac{c}{\beta}$ *and* $\lambda = 0$.

(iv) $x_{\mathrm{C}} + x_{\mathrm{OC}} + x_{\mathrm{OD}} = 1$ *and* $y_{\mathrm{SR}} = 1$, *where* $x_{\mathrm{C}} \leq 1 - \frac{\gamma}{b}$ *and* $\lambda = 1$.

(v) $x_{\mathrm{OC}} = 1$ *and* $y_{\mathrm{NR}} + y_{\mathrm{SR}} = 1$, *where* $y_{\mathrm{NR}} \leq 1 - \frac{c}{\beta}$ *and* $\lambda = \frac{\gamma}{b}$.

In this list of mixed Nash equilibria, the first case is qualitatively similar to the pure (*OC*, *SR*) equilibrium. Here, donors can mix (*OC*, *SR*) and (*C*, *SR*), provided that they do not engage in unconditional

cooperation too often (otherwise the recipient would no longer be sufficiently incentivized to engage in social rewarding). The second case is qualitatively new. It states that under appropriate conditions, donors may want to randomize between the two opportunistic strategies, while recipients randomize between non-rewarding and social rewarding. The remaining cases are again non-generic.

1.3 The case of mutually beneficial rewards

In our previous analysis we have assumed that rewarding is costly for the recipient, $\gamma > 0$. We have found that such rewards may be used in equilibrium if they incentivize the donor to cooperate. However, in some applications, providing the donor with a reward may be mutually beneficial, such that $\gamma < 0$. In that case, recipients may be incentivized to reward the donor even if the donor did defect. This in turn could undermine the incentivizing effect of rewards on cooperation. In fact, when $\gamma < 0$, the decision not to reward someone may be interpreted as an instance of costly punishment (because it leads to opportunity costs for both parties). Here, we thus explore the possible equilibria when rewards are mutually beneficial. The following is the respective analogue of Lemma 1.

Lemma 2. *Suppose the parameters satisfy* $0<c<b$ *and* $\gamma<0<\beta$.

1. *For all strategies* $\mathbf{y}$ *of the recipient, the donor's payoffs satisfy*

$$\pi_{\mathbf{DO}}(\mathrm{C}, \mathbf{y}) \leq \pi_{\mathbf{DO}}(\mathrm{OC}, \mathbf{y}) \quad \textit{and} \quad \pi_{\mathbf{DO}}(\mathrm{D}, \mathbf{y}) \leq \pi_{\mathbf{DO}}(\mathrm{OD}, \mathbf{y}). \tag{6}$$

   The first inequality is strict if and only if $\lambda>0$ *and* $y_{\mathrm{SR}}<1$. *The second inequality is strict if and only if* $\lambda>0$ *and* $y_{\mathrm{SR}}>0$.

2. *For all mixed strategies* $\mathbf{x}$ *of the donor, the recipient's payoffs satisfy*

$$\pi_{\mathbf{RE}}(\mathbf{x}, \mathrm{AR}) \leq \pi_{\mathbf{RE}}(\mathbf{x}, \mathrm{UR}) \quad \textit{and} \quad \pi_{\mathbf{RE}}(\mathbf{x}, \mathrm{NR}) < \pi_{\mathbf{RE}}(\mathbf{x}, \mathrm{UR}). \tag{7}$$

   The first inequality is strict if and only if $x_{\mathrm{C}}=1$ *(when* $\lambda=1$*) or* $x_{\mathrm{C}}+x_{\mathrm{OC}}=1$ *(when* $\lambda<1$*).*

The first part of this statement coincides with corresponding statement in Lemma 1. For donors, opportunistic behaviors remain advantageous. The second part, however, is different. Now it is never rewarding that is strictly dominated by unconditional rewarding. This result reflects the modified incentive structure when rewards are mutually beneficial: now, recipients have an intrinsic incentive to always reward the donor (whereas in the previous case, recipients had an incentive to avoid rewards when possible). As the following result shows, this modified incentive structure has a considerable impact on the set of Nash equilibria.

Proposition 4. *Suppose the parameters satisfy* $0 < c < b$ *and* $\gamma < 0 < \beta$, *and let* $(\mathbf{x}, \mathbf{y})$ *be a strategy profile. Then* $(\mathbf{x}, \mathbf{y})$ *is a pure Nash equilibrium if and only if one of the following cases applies:*

(i) $(\mathbf{x},\mathbf{y}) \in \Big\{(\mathrm{C},\mathrm{SR}),(\mathrm{OC},\mathrm{SR}),(\mathrm{D},\mathrm{AR}),(\mathrm{D},\mathrm{UR})\Big\}$,

(ii) $(\mathbf{x},\mathbf{y}) \in \Big\{(\mathrm{OD},\mathrm{AR}),(\mathrm{OD},\mathrm{UR})\Big\}$ *and* $\lambda \leq -\frac{\gamma}{b-\gamma}$,

(iii) $(\mathbf{x},\mathbf{y})$ = (OD, SR) *and* $\lambda = 1$.

Two remarks are in order. First, the above result suggests that even if recipients have strong incentives to reward anyone, there are still equilibria in which they selectively reward cooperators only. Second, once rewards are mutual beneficial, even antisocial rewarding may arise in equilibrium. However, in both of the above cases, we note that in equilibrium, recipients turn out to always reward their opponent, because donors appropriately adapt their behavior. When recipients engage in social rewarding, donors learn to always cooperate (either with *C* or *OC*). Conversely, when recipients engage in antisocial rewarding, donors always defect (either with *D* or *OD*).

2 Supplementary Note 2: Equilibrium analysis of the multiplayer game

2.1 Description of the game

To consider games among more than two players, we consider a group of size N. As before, the game proceeds in two stages. In the first stage, players decide whether or not to pay a cost $c > 0$ to contribute to a public good. Total contributions are multiplied with a factor of r and equally divided among all group members. Assuming that $1 < r < N$, the game is a social dilemma: everybody prefers their co-players to contribute to the public good, but at the same time everybody is incentivized to withhold one's own contributions. In the second stage, players can then decide whether or not to individually reward other group members. In the following, we will restrict our attention to rewards that are individually costly: the rewarding player pays a cost $\gamma > 0$ to offer a payoff of β to another group member.

With respect to the players' possible strategies, our setup follows the two-player model. As before, we consider four possible behaviors in the second stage:

(i) Never rewarding (reward no one; *NR*),

(ii) Social rewarding (reward all group members that contributed to the public good; *SR*),

(iii) Antisocial rewarding (reward all group members that did not contribute to the public good; *AR*),

(iv) Unconditional rewarding (reward everyone; *UR*).

For the first stage, we assume that with probability λ, all players know the rewarding strategies of all other group members; with the converse probability $\bar{\lambda} := 1 - \lambda$ they know nothing about the group composition. Under that assumption, we consider the following four behaviors for the first stage:

(i) Unconditional cooperation (always contribute to the public good; *C*),

(ii) Opportunistic cooperation (contribute if the strategy of the other group members is unknown; otherwise contribute if and only if doing so yields at least the payoff of non-contributing, given the known rewarding strategies of the other group members; *OC*),

(iii) Opportunistic defection (defect if the strategy of the other group members is unknown; otherwise contribute if and only if this yields at least the payoff than non-contributing, given the known rewarding strategies of the other group members; *OD*),

(iv) Unconditional defection (never contribute to the public good; *D*).

In contrast to the asymmetric two-player game studied before, now each player makes a decision in both stages. Hence, the pure strategies of the game take the form $\sigma = (\sigma_1, \sigma_2)$. Here, $\sigma_1 \in \{C, OC, OD, D\}$ determines the player's behavior in the first stage, and $\sigma_2 \in \{NR, SR, AR, UR\}$ determines the player's behavior in the second stage. In particular, there are sixteen pure strategies in total. In the following, we assume that individuals use pure strategies throughout.

To define payoffs, consider a player with strategy $\mathbf{s}$, and assume the player interacts in a group with composition

$$\mathbf{m} = (m_{(C,NR)},\ m_{(C,SR)},\ \ldots,\ m_{(D,UR)}),$$

where $m_{\sigma'}$ is the number of co-players with strategy σ'. Given the group composition $\mathbf{m}$, we can compute the corresponding marginal abundances

$$\begin{aligned} m_C &:= \sum_{\sigma_2} m_{(C,\sigma_2)}, & m_{OC} &:= \sum_{\sigma_2} m_{(OC,\sigma_2)}, & m_{OD} &:= \sum_{\sigma_2} m_{(OD,\sigma_2)}, & m_D &:= \sum_{\sigma_2} m_{(D,\sigma_2)}, \\ m_{NR} &:= \sum_{\sigma_1} m_{(\sigma_1,NR)}, & m_{SR} &:= \sum_{\sigma_1} m_{(\sigma_1,SR)}, & m_{AR} &:= \sum_{\sigma_1} m_{(\sigma_1,AR)}, & m_{UR} &:= \sum_{\sigma_1} m_{(\sigma_1,UR)}, \end{aligned} \tag{8}$$

These marginal abundances reflect how many of the co-players engage in each possible stage behavior. We can use these marginal abundances to rigorously define when an opportunistic player (with first-stage strategy *OC* or *OD*) would cooperate when the group composition is known. Such a player cooperates if and only if the expected surplus from rewards exceeds the effective cost of contributing,

$$\beta(m_{SR} - m_{AR}) \geq (1 - \frac{r}{N})c. \tag{9}$$

In particular, opportunists are more easily swayed to contribute when many group members use social rewarding. They are discouraged from contributing in groups with many anti-social rewarders.

Given the above considerations, the payoff $\pi_{\mathbf{m}}(\mathbf{s})$ of a player with strategy $\mathbf{s}$ in a group with composition $\mathbf{m}$ is well-defined. However, the respective formula is laborious to compute (as the first-stage behaviors of each group member might depend on the second-stage behavior of all other group members). Instead of providing an explicit formula, we provide the algorithm that we used to compute payoffs for our evolutionary simulations in our online repository.

Similar to the two-player case, one can show that rewards can only sustain cooperation if both the public good and rewards are sufficiently valuable. The following can thus be considered as the multiplayer version of Proposition 1.

Proposition 5. *Suppose that the effective cost of cooperation is not compensated by the maximum reward, or that the cost of rewarding is not compensated by the respective benefit of cooperation, such that parameters either satisfy*

$$\beta(N-1) < (1-\frac{r}{N})c \quad \text{or} \quad \frac{rc}{N} < \gamma. \tag{10}$$

Then there is no pure Nash equilibrium in which players contribute to the public good, or in which they reward other group members.

To allow for non-trivial results, in the following we thus assume that both rewards and contributions to the public good are sufficiently valuable, $(1-\frac{r}{N})c < \beta(N-1)$ and $\gamma < \frac{rc}{N}$.

2.2 Characterization of Nash equilibria

To describe the Nash equilibria of the multiplayer game, it is again useful to characterize which strategies are (weakly) dominated.

Lemma 3. *Suppose the parameters satisfy* $0 < (1-\frac{r}{N})c < \beta(N-1)$ *and* $0 < \gamma < \frac{rc}{N}$.

1. *For all group compositions* $\mathbf{m}$ *and all* $\sigma_2 \in \{\text{NR}, \text{SR}, \text{AR}, \text{UR}\}$, *payoffs satisfy*

$$\pi_{\mathbf{m}}(\text{C}, \sigma_2) \leq \pi_{\mathbf{m}}(\text{OC}, \sigma_2) \quad \text{and} \quad \pi_{\mathbf{m}}(\text{D}, \sigma_2) \leq \pi_{\mathbf{m}}(\text{OD}, \sigma_2). \tag{11}$$

   The first inequality is strict if and only if $\lambda > 0$ *and* $\beta(m_{\text{SR}} - m_{\text{AR}}) < (1-\frac{r}{N})c$.
   The second inequality is strict if and only if $\lambda > 0$ *and* $\beta(m_{\text{SR}} - m_{\text{AR}}) > (1-\frac{r}{N})c$.

2. *For all group compositions* $\mathbf{m}$ *and all* $\sigma_1 \in \{\text{C}, \text{OC}, \text{OD}, \text{D}\}$, *payoffs satisfy*

$$\pi_{\mathbf{m}}(\sigma_1, \text{AR}) \leq \pi_{\mathbf{m}}(\sigma_1, \text{NR}) \quad \text{and} \quad \pi_{\mathbf{m}}(\sigma_1, \text{UR}) < \pi_{\mathbf{m}}(\sigma_1, \text{NR}). \tag{12}$$

   The first inequality is strict if and only if, given the group composition $\mathbf{m}$, *one of the following two cases applies. (i) There is a positive probability that some other group member defects. (ii) There is a positive probability that some opportunistic contributors among the other group members would cooperate if only the focal player did not engage in antisocial rewarding.*

The interpretation of this result is similar to the interpretation of the corresponding Lemma 1 for the two-player game, with one exception. In the two-player game, antisocial rewarding is only strictly dominated when it leads the focal player to pay a rewarding cost. In the multiplayer game, antisocial rewarding is

additionally dominated when it deters some of the other group members from contributing. Based on these preparations, we can now characterize the symmetric Nash equilibria of the game (i.e., the Nash equilibria in which all group members adopt the same strategy).

Proposition 6. *Suppose parameters satisfy* $0<(1-\frac{r}{N})c<\beta(N-1)$ *and* $0<\gamma<\frac{rc}{N}$*, and let* $\sigma=(\sigma_1,\sigma_2)$ *be a pure strategy. Then* σ *is a (symmetric) Nash equilibrium if and only if one of the following four cases applies:*

(i) $\sigma=(\mathrm{D},\mathrm{NR})$.

(ii) $\sigma=(\mathrm{OC},\mathrm{SR})$ *and both,* $\beta<(1-\frac{r}{N})\frac{c}{N-2}$ *and* $\lambda\geq\frac{N}{rc}\gamma$.

(iii) $\sigma=(\mathrm{OD},\mathrm{SR})$ *and both,* $\beta<(1-\frac{r}{N})\frac{c}{N-2}$ *and* $\lambda=1$.

(iv) $\sigma=(\mathrm{OD},\mathrm{NR})$ *and either* $\lambda=0$ *or* $\beta\leq(1-\frac{r}{N})c$.

(v) $\sigma=(\mathrm{OC},\mathrm{NR})$ *and both,* $\lambda=1$ *and* $\beta<c(1-\frac{r}{N})$.

Qualitatively, again this set of pure Nash equilibria agrees with the corresponding set in the two-player game. There are two major cases. In one case, group members neither contribute to the public good, nor do they reward each other. In the other case, they all contribute to the public good and engage in social rewarding. However, there is one major difference. For a homogeneous (OC,SR) group to be a Nash equilibrium, rewards must not be too profitable, $\beta<(1-\frac{r}{N})\frac{c}{N-2}$. Once rewards are too profitable, opportunistic group members find it worth to contribute even if not all other group members engage in social rewarding. As a result, a second-order free riding problem arises: individuals understand that it takes some social rewarding to ensure mutual cooperation, but they prefer others to pay the respective rewarding costs. Evolutionary simulations with positive mutation rates suggest that this can lead the coexistence of socially-rewarding and non-rewarding cooperators (see **Fig. 4d**). The following result describes when such a coexistence can occur.

Proposition 7. *Suppose parameters satisfy* $0<(1-\frac{r}{N})c<\beta(N-1)$ *and* $0<\gamma<\frac{rc}{N}$*. Consider a group in which* n_{SR} *players adopt (*OC, SR*) and* $N-n_{\mathrm{SR}}$ *players adopt (*OC, NR*), with* $0<n_{\mathrm{SR}}<N$*. This group composition gives rise to an (asymmetric) Nash equilibrium in which all players contribute if and only if*

$$\max\left(\big(1-\frac{r}{N}\big)\frac{c}{\beta},\ (N-1)\frac{N}{rc}\frac{\gamma}{\lambda}\right)\leq n_{\mathrm{SR}}-1<1+\big(1-\frac{r}{N}\big)\frac{c}{\beta}. \tag{13}$$

The intuition for Proposition 7 is as follows. To sustain cooperation in an asymmetric equilibrium, in which individuals cooperate opportunistically, and in which some individuals reward socially whereas others do not, two requirements need to hold. First, the number of socially rewarding group members n_{SR} needs to hit a sweet spot to incentivize cooperation. There need to be sufficiently many such that all group members find it worthwhile to cooperate. Yet there must not be more than necessary. This first requirement is reflected in the terms that involve c/β. Second, the public good contributions of the

remaining $n_{SR}-1$ social rewarders need to be sufficiently valuable compared to the cost of rewarding all $N-1$ group members for their cooperation. This second requirement is reflected by the term that involves γ/λ.

3 Supplementary Note 3: Proofs

3.1 Proofs for the two-player game

Proof of Proposition 1. Let $(\mathbf{x}, \mathbf{y})$ be a Nash equilibrium. Moreover, we assume in the following that the information transmissibility is intermediate, such that $0 < \lambda < 1$ (the boundary cases $\lambda \in \{0,1\}$ follow similarly). The proof is based on the observation that in a Nash equilibrium, players do not use dominated strategies[2].

1. If $\beta < c$, it follows from Eq. (1) that both *C* and *OC* are strictly dominated by *D*. Therefore, $x_C + x_{OC} = 0$, or equivalently, $x_{OD} + x_D = 1$. Given the donor's behavior, it follows from Eq. (2) that recipients strictly prefer *NR* to both *AR* and *UR*, i.e., $\pi_{\mathbf{RE}}(\mathbf{x}, NR) > \pi_{\mathbf{RE}}(\mathbf{x}, AR)$ and $\pi_{\mathbf{RE}}(\mathbf{x}, NR) > \pi_{\mathbf{RE}}(\mathbf{x}, UR)$. Therefore, $y_{NR}+y_{SR}=1$. Finally, we note that when $y_{SR} > 0$, then donors strictly prefer to defect unconditionally, $\pi_{\mathbf{DO}}(D, \mathbf{y}) > \pi(OD, \mathbf{y})$. Overall, we obtain that $(\mathbf{x}, \mathbf{y})$ can only be a Nash equilibrium if one of the following two cases applies,

$$\begin{aligned} x_D = 1 \ \text{ and } \ y_{NR} + y_{SR} = 1 \\ x_{OD} + x_D = 1 \ \text{ and } \ y_{NR} = 1 \end{aligned} \tag{14}$$

   We note that in both cases, donors never cooperate, and therefore recipients never reward (moreover, we note that $x_{OD} > 0$ can only occur in equilibrium if in addition $b < \gamma$ holds).

2. If $b < \gamma$, again *UR* is strictly dominated by *NR*. Moreover, *AR* is weakly dominated, $\pi_{\mathbf{RE}}(\mathbf{x}, AR) \leq \pi_{\mathbf{RE}}(\mathbf{x}, NR)$, with equality if and only if $x_C = 1$. We note that the case $x_C = 1$ and $y_{AR} > 0$ cannot arise in equilibrium, because in that case, cooperators could do strictly better by choosing opportunistic cooperation instead. Therefore, again $y_{NR} + y_{SR} = 1$. If $y_{NR} = 1$, donors are indifferent between *OD* and *D*. If, on the other hand, $y_{NR} < 1$, then $x_D = 1$, for otherwise social rewarders could do strictly better by switching to non-rewarding. Again, we conclude that the Nash equilibrium $(\mathbf{x}, \mathbf{y})$ needs to satisfy one of the two cases in (14) (here, we note that $y_{SR} > 0$ can only occur in equilibrium if in addition $\beta < c$ holds).

□

Proof of Lemma 1. The first part follows directly from the payoff matrix depicted in Eq. (1), by comparing the respective payoff entries in the corresponding rows. Similarly, the second part follows directly from the payoff matrix (2), by comparing the respective columns. □

Proof of Proposition 2. We prove the statement by considering all possible strategies for the recipient. By Lemma 1, it follows that *UR* cannot be played in any Nash equilibrium. Similarly, it also follows

that *AR* cannot be played in a Nash equilibrium – if there was a Nash equilibrium of the form $(\mathbf{x}, AR)$, it would follow from Eq. (7) that the donor's strategy needs to be *C*, but *C* is not a best response to *AR*.

Now, suppose the recipient's strategy is *SR*. By requiring that the donor plays a best response, it follows from Eq. (1) that there are two equilibrium candidates (*C*, *SR*), (*OC*, *SR*); in addition, (*OD*, *SR*) is an equilibrium candidate if we additionally require that $\lambda = 1$. The first equilibrium candidate (*C*, *SR*) can be ruled out, because for recipients, *SR* is not a best response to an unconditionally cooperative donor. The second equilibrium candidate (*OC*, *SR*) is a Nash equilibrium if also the recipient has no incentive to deviate. From Eq. (2) it follows that this requirement is true if and only if $\bar{\lambda} b \leq b - \gamma$, or equivalently, $\lambda \geq \frac{\gamma}{b}$. Finally, the third equilibrium candidate (*OD*, *SR*) is indeed an equilibrium, provided that $\lambda = 1$. This corresponds to the cases (*ii*) and (*iii*).

Finally, suppose the recipient's strategy is *NR*. Again by requiring the donor to play a best response, we obtain the two equilibrium candidates (*D*, *NR*) and (*OD*, *NR*). By also requiring the recipient to choose a best response, it follows from Eq. (2) that (*D*, *NR*) is always a Nash equilibrium, whereas (*OD*, *NR*) is only a Nash equilibrium if $\lambda = 0$. This corresponds to the cases (*i*) and (*iv*). □

Proof of Proposition 3. Similar to the proof of Proposition 2, we prove this result by considering all possible strategies that the recipient may play in equilibrium with positive probability. As before, we can rule out *UR* and *AR*. Therefore, any Nash equilibrium $(\mathbf{x}, \mathbf{y})$ must satisfy $y_{NR} + y_{SR} = 1$. This leaves us with the following three cases:

$\underline{y_{SR} = 1}$. Again by requiring that the donor play a best response, we conclude that $(\mathbf{x}, SR)$ can only be an equilibrium if either $x_C + x_{OC} = 1$ (if $\lambda < 1$), or if $x_C + x_{OC} + x_{OD} = 1$ (if $\lambda = 1$). In the first case, *SR* is a best response to the donor's mixed strategy if and only if $bx_C + \bar{\lambda} b x_{OC} \leq b - \gamma$, which is equivalent to $x_C \leq 1 - \frac{\gamma}{\lambda b}$. In the second case (with $\lambda = 1$), *SR* is a best response if and only if $bx_C \leq b - \gamma$, which is equivalent to $x_C \leq 1 - \frac{\gamma}{b}$. This corresponds to the cases (*i*) and (*iv*).

$\underline{y_{SR} = 0}$. By requiring the donor to best respond, it follows that all possible equilibrium candidates $(\mathbf{x}, NR)$ need to satisfy either $x_{OD} + x_D = 1$ (if $\lambda < 1$) or $x_{OC} + x_{OD} + x_D = 1$ (if $\lambda = 1$). In the first case, by requiring that *NR* is a best response to the mixed strategy $\mathbf{x}$, we conclude that either $x_{OD} = 0$ (which would result in a pure Nash equilibrium) or $\lambda = 0$. In the second case, $x_{OC} > 0$ is ruled out because then, *NR* would no longer be a best response to $\mathbf{x}$. This corresponds to one special case of (*iii*).

$\underline{0 < y_{SR} < 1}$. For this case, let us first assume that $\lambda > 0$. In that case, it follows from Lemma 1 that *C* yields a strictly lower payoff than *OC*, and that *D* yields a strictly lower payoff than *OD*. Therefore, by requiring that donors best respond to $\mathbf{y}$, we obtain that $(\mathbf{x}, \mathbf{y})$ needs to satisfy $x_{OC} + x_{OD} = 1$. In order for both *NR* and *SR* to be sustained in equilibrium, recipients need to be indifferent, which yields the necessary condition $\bar{\lambda} b x_{OC} = (b - \gamma) x_{OC} + \lambda (b - \gamma) x_{OD}$. This in turn implies $x_{OC} = \frac{\lambda(b-\gamma)}{(1-\lambda)\gamma}$ and $x_{OD} = 1 - \frac{\lambda(b-\gamma)}{(1-\lambda)\gamma}$. To determine the possible values of y_{NR} and y_{SR}, we again need to distinguish three cases:

$\underline{x_{OC}=1.}$ In that case, y_{NR} and y_{SR} need to be chosen such that *OC* is indeed a best response to $\mathbf{y}$. This implies that $-\bar{\lambda} c y_{NR} + (\beta - c) y_{SR} \geq \lambda(\beta - c) y_{SR}$, which in turn yields the condition $y_{NR} \leq 1 - \frac{c}{\beta}$.

$\underline{x_{OD}=0.}$ This case can only occur when $\lambda = 0$, which we have ruled out by assumption.

$\underline{0 < x_{OD} < 1.}$ In that case, y_{NR} and y_{SR} need to be chosen such that donors are indifferent between *OC* and *OD*. This leads to the condition $-\bar{\lambda} c y_{NR} + (\beta - c) y_{SR} = \lambda(\beta - c) y_{SR}$ or $y_{NR} = 1 - \frac{c}{\beta}$ and $y_{SR} = \frac{c}{\beta}$.

This corresponds to the cases (*ii*) and (*v*).

Finally, for $\lambda = 0$, it follows from Eq. (2) that recipients are only indifferent between *NR* and *SR* if $x_{OD} + x_D = 1$. For *OD* and *D* to be best responses to the recipient's strategy, in turn it needs to be the case that $y_{NR} > 1 - \frac{c}{\beta}$. This corresponds to case (*iii*). □

Proof of Lemma 2. Similar to the proof of Lemma 1, the result follows directly from the respective payoff matrices (1) and (2), by taking into account that now $\gamma < 0$. □

Proof of Proposition 4. Again, we prove the Proposition by considering all possible strategies of the recipient. By Lemma 2, *NR* cannot be sustained in a Nash equilibrium.

If the recipient adopts the strategy *SR*, both *C* and *OC* are best responses for the donor. In addition, *OD* is a best response if $\lambda = 1$. Conversely, if the donor adopts one of these strategies, it follows from payoff matrix (2) that *SR* is indeed a best response to all these strategies.

If the recipient adopts the strategy *AR*, the donor's best response is either *OD* or *D*. Conversely, if the donor adopts *D*, recipients have no incentive to deviate from *AR*. If the donor instead adopts *OD*, recipients do not benefit from deviating if and only if $\lambda(b - \gamma) \leq -\gamma$, or equivalently $\lambda \leq -\frac{\gamma}{b-\gamma}$.

Finally, if the recipient adopts the strategy *UR*, both *D* and *OD* are best responses. In addition, *OD* is a best response if $\lambda = 1$. Conversely, if the donor adopts *D*, the recipient cannot do better than choosing *UR*. If the donor adopts *OD*, the recipient cannot do better provided that $\lambda \leq -\frac{\gamma}{b-\gamma}$. Finally, if $\lambda = 1$ and the donor chooses *OC*, the recipient prefers to deviate from *UR*, by switching to *SR*. Hence, (*D*, *UR*) and (*OD*, *UR*) are the only possible Nash equilibria that feature unconditional rewarding. □

3.2 Proofs for the multiplayer game

Proof of Proposition 5. The proof follows the lines of the proof of the two-player version in Proposition 1. Suppose that $\sigma = (\sigma_1, \sigma_2)$ is a Nash equilibrium in pure strategies, and suppose that $0 < \lambda < 1$ (again, the proof for $\lambda \in \{0, 1\}$ is similar, and is therefore omitted. We consider the two cases separately.

<u>Rewards are not sufficiently profitable, $\beta(N-1) < (1 - \frac{r}{N})c$.</u>

First, we note that for any group composition $\mathbf{m}$, any strategy of the form (C, σ_2) is strictly domi-

nated by (D, σ_2), because

$$\pi_{\mathbf{m}}(C, \sigma_2) - \pi_{\mathbf{m}}(D, \sigma_2) = -(1-\frac{r}{N})c + (m_{SR} - m_{AR})\beta \leq -(1-\frac{r}{N})c + (N-1)\beta < 0. \quad (15)$$

For (OC, σ_2), we obtain an analogous result. Note that by assumption, Eq. (9) is never satisfied, and hence opportunists defect when they know the group composition. As a consequence,

$$\pi_{\mathbf{m}}(OC, \sigma_2) - \pi_{\mathbf{m}}(D, \sigma_2) = \bar{\lambda} \cdot \big[-(1-\frac{r}{N})c + (m_{SR} - m_{AR})\beta \big] < 0. \quad (16)$$

Therefore, any pure Nash equilibrium (σ_1, σ_2) needs to satisfy $\sigma_1 \in \{OD, D\}$; in both cases, players never cooperate. Similarly, we can also show that no player engages in rewarding. Indeed, it is easy to see that given $m_D + m_{OD} = N-1$, both antisocial rewarding and unconditional rewarding yield a strictly lower payoff than never rewarding,

$$\pi_{\mathbf{m}}(\sigma_1, UR) - \pi_{\mathbf{m}}(\sigma_1, NR) = \pi_{\mathbf{m}}(\sigma_1, AR) - \pi_{\mathbf{m}}(\sigma_1, NR) = -(N-1)\gamma < 0 \quad \text{for all } \sigma_1. \quad (17)$$

As a result, any pure Nash equilibrium needs to be of the form (*D*, *NR*), (*OD*, *NR*), (*D*, *SR*), (*OD*, *SR*). In all these equilibria, no one cooperates and no one rewards.

Public good contributions are not sufficiently profitable, $rc/N < \gamma$.

Given the assumption, it is easy to show that no player will reward in equilibrium. Indeed, any strategy that involves unconditional rewarding is strictly dominated because

$$\pi_{\mathbf{m}}(\sigma_1, UR) - \pi_{\mathbf{m}}(\sigma_1, NR) \leq -(N-1)\gamma < 0 \quad \text{for all } \sigma_1. \quad (18)$$

Similarly, antisocial rewarding is weakly dominated, and the dominance is strict if any of the group members has a positive probability of defecting. Finally, for social rewarding we get

$$\pi_{\mathbf{m}}(\sigma_1, SR) - \pi_{\mathbf{m}}(\sigma_1, NR) \leq (m_C + \bar{\lambda} m_{OC})(\frac{r}{N} - \gamma) \leq 0 \quad \text{for all } \sigma_1. \quad (19)$$

The second-to-last inequality indicates that social rewarding has the advantage of possibly swaying some group members to cooperate; however, this advantage is outweighed by the respective rewarding costs γ. Moreover, (σ_1, SR) yields a strictly lower payoff than (σ_1, NR) if any co-player has a positive probability of cooperating. Taking into account the above considerations, we obtain the equilibrium candidates (*D*, *NR*), (*OD*, *NR*), (*D*, *SR*), (*OD*, *SR*), (*C*,*AR*). However, the last strategy profile can be ruled out because players have an incentive to deviate from *C*. Moreover, the strategy profiles (*D*, *SR*), (*OD*, *SR*) can be ruled out if $\beta(N-1) \geq (1-\frac{r}{N})c$. We conclude that the only equilibrium candidates are (*D*, *NR*), (*OD*, *NR*), and (*D*, *SR*), (*OD*, *SR*) if additionally $\beta(N-1) < (1-\frac{r}{N})c$. In all these cases no player cooperates, and no player rewards any co-player. □

Proof of Lemma 3. 1. In this part of the proof it is shown that playing a conditional donor strategy (*OC* or *OD*) dominates playing the corresponding unconditional donor strategy (*C* or *D*) given the same play of the rewarding strategy, i.e. Eq. (11) and Eq. (12) when the parameters satisfy $0 < (1-\frac{r}{N})c < \beta(N-1)$ and $0 < \gamma < \frac{rc}{N}$. For this we need to consider two cases:

Rewards are sufficiently profitable: $\beta(m_{SR} - m_{AR}) \geq c(1-\frac{r}{N})$:

In this case, unconditional cooperators and opportunistic cooperators behave exactly the same way, given they both use the same rewarding strategy. Therefore, they receive the same payoffs given the same co-player composition. That is, for all possible group composition $\mathbf{m}$ and $\sigma_2 \in \{NR, SR, AR, UR\}$:

$$\pi_{\mathbf{m}}(C, \sigma_2) = \pi_{\mathbf{m}}(OC, \sigma_2) \tag{20}$$

Let us denote with T the total contribution made by the co-players to the public good for a group composition $\mathbf{m}$. The payoffs earned by playing the strategy (D, σ_2) is then:

$$\pi_{\mathbf{m}}(D, \sigma_2) = \frac{rT}{N} + \beta(m_{AR} + m_{UR}) - \Gamma \tag{21}$$

And the payoff earned by a (OD, σ_2) player is:

$$\pi_{\mathbf{m}}(OD, \sigma_2) = \frac{r\lambda c}{N} + \frac{rT}{N} - \lambda c + \lambda\beta m_{SR} + (1-\lambda)\beta m_{AR} + \beta m_{UR} - \Gamma \tag{22}$$

Where Γ is the total cost that the focal player with the rewarding strategy σ_2 has to pay for rewarding their $N-1$ co-players. Note that the total rewarding cost, Γ and the total contribution made by the focal player's co-players to the public good, T, remain the same irrespective of whether the focal player plays (D, σ_2) or (OD, σ_2). Thus, the difference in their payoffs:

$$\pi_{\mathbf{m}}(OD, \sigma_2) - \pi_{\mathbf{m}}(D, \sigma_2) = \lambda(\beta(m_{SR} - m_{AR}) - c(1 - \frac{r}{N})) \geq 0 \tag{23}$$

for all values of $0 \leq \lambda \leq 1$. The inequality is strict when both $\lambda > 0$ and $\beta(m_{SR} - m_{AR}) > c(1-\frac{r}{N})$.

Rewards are not sufficiently profitable: $\beta(m_{SR} - m_{AR}) < c(1-\frac{r}{N})$:

In this case, unconditional and conditional defectors, with the same rewarding strategy σ_2, behave identically in both the donating and the rewarding stage. The co-players' donation to the public good does not alter depending on whether *D* or *OD* is being played by the focal player. Therefore the payoff received by the strategies (D, σ_2) and (OD, σ_2) are the same. That is, for all possible group composition $\mathbf{m}$ and $\sigma_2 \in \{NR, SR, AR, UR\}$:

$$\pi_{\mathbf{m}}(D, \sigma_2) = \pi_{\mathbf{m}}(OD, \sigma_2) \tag{24}$$

When rewards are not sufficiently profitable, unconditional cooperators and opportunistic coop-

erators with the same strategy, no longer behave identically in the two stages. Using the same notations as before, the payoff for a (C, σ_2) player, who now cooperates with probability 1 is:

$$\pi_{\mathbf{m}}(C, \sigma_2) = \frac{r(T+c)}{N} - c + \beta(m_{SR} + m_{UR}) - \Gamma \tag{25}$$

While the payoff for a (OC, σ_2), who cooperates with probability $1 - \lambda$ in the same group composition is:

$$\pi_{\mathbf{m}}(OC, \sigma_2) = \frac{r(T+(1-\lambda)c)}{N} - (1-\lambda)c + \beta m_{UR} + (1-\lambda)\beta m_{SR} + \lambda\beta m_{AR} - \Gamma \tag{26}$$

The difference in their payoffs indicate that:

$$\pi_{\mathbf{m}}(OC, \sigma_2) - \pi_{\mathbf{m}}(C, \sigma_2) = \lambda(c(1-\frac{r}{N}) - \beta(m_{SR}-m_{AR})) \geq 0 \tag{27}$$

for all values in $0 \leq \lambda \leq 1$ and all group compositions $\mathbf{m}$. This inequality is strict when both $\lambda > 0$ and $\beta(m_{SR}-m_{AR}) < c(1-\frac{r}{N})$.

2. In this part of the proof it is shown that for a group composition $\mathbf{m}$ and a pure donating strategy σ_1, never rewarding dominates the pure rewarding strategies antisocial rewarding (weakly) and unconditional rewarding (strictly), i.e. Eq (12).
Let the total contribution from the co-players in the composition $\mathbf{m}$ be T when focal player plays (σ_1, NR). Let, us assume that the focal player contributes to the public goods with a probability p_f. Note that $p_f \in \{0, \lambda, 1-\lambda, 1\}$ since we are only considering pure strategies. Then,

$$\pi_{\mathbf{m}}(\sigma_1, NR) = \frac{rT+rp_f c}{N} - p_f c + \beta m_{UR} + \beta p_f m_{SR} + \beta(1-p_f)m_{AR} \tag{28}$$

When the focal player is playing a strategy (σ_1, UR) with the same group composition $\mathbf{m}$, the total contribution by co-players, T, the contribution by focal player to the public goods, $p_f c$, and the rewards gained, $\beta m_{UR} + \beta p_f m_{SR} + \beta(1-p_f)m_{AR}$, are exactly the same to the case when the focal player plays (σ_1, NR). Only now, the focal player has to unconditionally reward all of their $N-1$ co-players. Therefore,

$$\pi_{\mathbf{m}}(\sigma_1, UR) - \pi_{\mathbf{m}}(\sigma_1, NR) = (N-1)\gamma > 0 \tag{29}$$

Let the total contribution from the co-players with composition $\mathbf{m}$ when the focal player is playing (σ_1, AR) be T'. Note that $T \geq T'$. This is because with an additional antisocial rewarder as their co-player (the focal player), only the opportunistic donors among the co-players may shift from cooperation to defection. Every other co-players' donation would not change. At most, the total contribution from the co-players can match T. The contribution from the focal individual

with the pure donor strategy σ_1, $p_f c$, is the same as before because it is dependent on the co-player composition $\mathbf{m}$ (which has not altered) and σ_1. The reward received on playing (σ_1, AR) is the same as the reward received on playing (σ_1, NR) against the play of $\mathbf{m}$. The antisocially rewarding focal player, however, now, has to pay the cost of rewarding for rewarding the *D* and *OD* co-players. Let this cost of rewarding be Γ. Therefore,

$$\pi_{\mathbf{m}}(\sigma_1, NR) - \pi_{\mathbf{m}}(\sigma_1, AR) = \frac{rc}{N}(T - T') + \Gamma \geq 0 \tag{30}$$

The above inequality is strict unless both $\Gamma = 0$ and $T = T'$. The term Γ is 0 when among all the co-players none of them play defect with a positive probability. The terms T and T' are equal only when by switching from *AR* to *NR*, none of the other group members start to cooperate. □

Proof of Proposition 6. We begin the characterization of all the symmetric pure strategy Nash Equilibria (σ_1, σ_2) of the multiplayer game by eliminating all the pure strategies that cannot be a Nash equilibrium. These can either be because they are strictly dominated by some other pure strategy or because a beneficial unilateral deviation to another pure strategy is possible.

From the statement of Lemma 3, it can be inferred that the strategies (σ_1, UR), where $\sigma_1 \in \{C, OC, OD, D\}$, cannot be a Nash equilibrium because they are strictly dominated by (σ_1, NR). Similarly, for all $\sigma_1 \in \{OC, OD, D\}$, the strategies (σ_1, AR) cannot be a Nash equilibrium because they are strictly dominated by (σ_1, NR). The only remaining pure strategy involving antisocial rewarding, (C, AR), is not an equilibrium since $\pi_{\mathbf{m}}(C, AR) < \pi_{\mathbf{m}}(D, AR)$ where group composition $\mathbf{m}$ is such that $m_{(C,AR)} = N-1$. Therefore, pure strategies of the form (σ_1, AR) and (σ_1, UR) cannot be a Nash equilibrium.

The strategy (C, NR) is not a Nash equilibrium because a deviation to (D, NR) is strictly better when all co-players play (C, NR). This is because the effective cost of cooperation, $c(1 - \frac{r}{N}) > 0$. The defector can gain by not contributing and earning a payoff through the contribution of the remaining $N-1$ unconditional cooperators. Similarly, the strategy (C, SR) is not a Nash equilibrium because a deviation to (C, NR) is strictly better as $\gamma > 0$ when all co-players play (C, NR). A never rewarding cooperator earns the same through the public goods and rewards from co-players but saves the cost of rewarding his cooperating co-players.

In a group composition where all co-players play (D, SR), the strategy (D, SR) earns the payoff 0. A deviation to (OC, SR) earns the player $-c(1 - \frac{r}{N}) + (N-1)\beta$. This payoff is strictly than 0 because of our standing assumption about the parameters. Therefore, (D, SR) is also not a Nash equilibrium.

1. <u>(D, NR) is an equilibrium:</u>
   The strategy (D, NR) earns a payoff 0 when all co-players play (D, NR). Any deviation to a pure rewarding strategy will earn a lower (or equal) payoff than 0 since the co-players are unconditional defectors and hence would never contribute to the pool, irrespective of group composition. Any deviation in the donor strategy would earn a payoff lower (or equal) to zero since (i) they would never be rewarded by co-players (ii) effective cost of cooperation by a single cooperator when all co-players are defectors: $c(1 - \frac{r}{N}) > 0$. Therefore, (D, NR) is a Nash equilibrium.

2. <u>Conditions for (OC, SR) to be an equilibrium:</u>

The payoff earned by playing (OC, SR) when all co-players are playing the same given that rewards are higher than the threshold that allows opportunists to cooperate when every co-player rewards socially: $\beta > \frac{c}{(N-1)}(1-\frac{r}{N})$ is:

$$\pi_{\mathbf{m}}(OC, SR) = c(r-1) + (N-1)(\beta-\gamma) \tag{31}$$

Where, $m_{(OC,SR)} = N-1$. We only focus on the cases where $\lambda > 0$ because at $\lambda = 0$, (OC, SR) is exactly the same as (C, SR) and we have demonstrated before that (C, SR) cannot be a Nash equilibrium. Now, let us assume that rewards are sufficiently high such that: $\beta(N-2) \geq c(1-\frac{r}{N})$. In this case, it is easy to see that a deviation from (OC, SR) to (OC, NR) can provide a strictly positive payoff difference. The deviated strategy will earn: $c(r-1) + (N-1)\beta$. Therefore, when $\beta \geq \frac{c}{N-2}(1-\frac{r}{N})$, (OC, SR) cannot be a Nash equilibrium.

Now, let us consider the case when $\frac{c}{N-1}(1-\frac{r}{N}) < \beta < \frac{c}{N-2}(1-\frac{r}{N})$. In this case too, a deviation to (OC, NR) can be strictly better if cost of rewarding is too high. The payoff difference upon deviation in this case is:

$$\pi_{\mathbf{m}}(OC, NR) - \pi_{\mathbf{m}}(OC, SR) = \lambda(N-1)(\gamma - \frac{rc}{N}) \tag{32}$$

Therefore, only when the cost of rewarding $\gamma \leq \frac{rc}{N}$ and $\beta(N-2) < c(1-\frac{r}{N})$, there is a possibility that (OC, SR) might be a Nash equilibrium. So, from here on, we focus on the following ranges of the parameters: $\frac{c}{N-1}(1-\frac{r}{N}) < \beta < \frac{c}{N-2}(1-\frac{r}{N})$ and $0 < \gamma \leq \frac{rc}{N}$ and $\lambda > 0$.

It has already been demonstrated that in these parameter ranges, a deviation towards (OC, NR) is never better in payoff. Now, let us consider a deviation towards (C, NR). This results in:

$$\pi_{\mathbf{m}}(OC, SR) - \pi_{\mathbf{m}}(C, NR) = (N-1)\big(\frac{\lambda rc}{N} - \gamma\big) \tag{33}$$

The deviation is strictly better if $\lambda < \frac{\gamma N}{rc}$. So, we focus on an even smaller parameter range: $\lambda \geq \frac{\gamma N}{rc}$ where it is still possible that (OC, SR) might be a Nash equilibrium – a low value of λ will allow a strictly better deviation towards the strategy (C, NR). Under these parameter ranges, a deviation towards (D, NR) earns a payoff difference of:

$$\pi_{\mathbf{m}}(OC, SR) - \pi_{\mathbf{m}}(D, NR) = (N-1)\big(\frac{\lambda rc}{N} - \gamma\big) + (N-1)\beta - c(1-\frac{r}{N}) \geq 0 \tag{34}$$

Therefore, deviation towards any pure strategy of the form (σ_1, NR) from (OC, SR) in a group composition where co-player plays (OC, SR) is never better given the parameters are in their respective ranges: $\frac{c}{N-1}(1-\frac{r}{N}) < \beta < \frac{c}{N-2}(1-\frac{r}{N})$ and $0 < \gamma \leq \frac{rc}{N}$ and $\lambda \geq \frac{\gamma N}{rc}$.

Now, consider deviations towards the strategies (σ_1, AR) or (σ_1, UR). The change in contributing

behaviour by the co-players when a deviation occurs from (OC, SR) to (σ_1, AR) or (σ_1, UR) is exactly the same as when a deviation occurs from (OC, SR) to (σ_1, NR). This is because we are interested in the very specific range of β where $N-1$ social rewarding co-players are enough to prompt cooperation from opportunistic cooperators but $N-2$ social rewarding co-players are not sufficient. The payoffs received (or not received), when such a deviation to (σ_1, AR) or (σ_1, UR) is made is exactly the same as when a deviation to (σ_1, NR) is made. The only difference with the case where the deviation was made to (σ_1, NR) is that now, a deviation to *UR* or *AR* would cost the deviating player the price of rewarding all the (or atleast all the defecting) co-players. Therefore, in this parameter regime:

$$\pi_{\mathbf{m}}(OC, SR) - \pi_{\mathbf{m}}(\sigma_1, NR) \geq 0 \implies \pi_{\mathbf{m}}(OC, SR) - \pi_{\mathbf{m}}(\sigma_1, \sigma_2) \geq 0 \tag{35}$$

where $\sigma_2 \in \{AR, UR\}$.

Lastly, we need to check deviations to pure strategies of the form (σ_1, SR) in this parameter regime. When the deviation is towards (C, SR), the payoff difference is exactly 0. When the deviation is towards (D, SR), the payoff difference is strictly greater than zero, as:

$$\pi_{\mathbf{m}}(OC, SR) - \pi_{\mathbf{m}}(D, SR) = -c(1-\frac{r}{N}) + (N-1)\beta > 0 \tag{36}$$

Finally, if the deviation is towards (OD, SR) the payoff difference is greater than 0 for all values of $\lambda > \frac{\gamma N}{rc}$. That is:

$$\pi_{\mathbf{m}}(OC, SR) - \pi_{\mathbf{m}}(OD, SR) = (1-\lambda)(-c(1-\frac{r}{N}) + (N-1)\beta) \geq 0 \tag{37}$$

Therefore, in this parameter regime, no deviation to (σ_1, SR) from (OC, SR) is better.

As all the possible deviations have been checked, and it was found that no deviation to a pure strategy yield greater payoff (under the parameter regime), it can be concluded that (OC, SR) is a Nash equilibrium when the following hold: $\beta < \frac{c}{N-2}(1 - \frac{r}{N})$ and $\lambda \geq \frac{\gamma N}{rc}$ (along with the standing assumptions).

3. Conditions for (OD, SR) to be an equilibrium:

   Now, let us consider the strategy (OD, SR). The payoff of that strategy when all co-players play the same strategy is: $\lambda c(r-1) + \lambda(N-1)(\beta - \gamma)$. All players cooperate and reward with probability λ as rewards are sufficiently high for opportunistic defectors: $(N-1)\beta > c(1 - \frac{r}{N})$. A deviation

to the strategy (C, SR) earns the player a payoff difference of:

$$\begin{aligned}\pi_{\mathbf{m}}(C,SR)-\pi_{\mathbf{m}}(OD,SR)=&\frac{\lambda(N-1)rc+cr}{N}-c+(N-1)\beta-\lambda(N-1)\gamma\\&-\lambda c(r-1)-\lambda(N-1)(\beta-\gamma)\\=&(1-\lambda)((N-1)\beta-c(1-\frac{r}{N}))\end{aligned}$$

Since we assume that $(N-1)\beta - c(1-\frac{r}{N}) > 0$, deviation to (C, SR) is strictly better when $\lambda < 1$. At $\lambda = 1$, the strategy (OD, SR) is identical to (OC, SR) and therefore the conditions evaluated for (OC, SR) to be an equilibrium apply here. That is, (OD, SR) is an equilibrium only when $\lambda = 1$ and $\beta < (1 - \frac{r}{N})\frac{c}{N-2}$.

4. Conditions for (OD, NR) to be an equilibrium:

   The payoff earned by a (OD, NR) player when all of his co-players are playing the same strategy is 0. Now, let us consider a deviation to (OD, SR). This deviation may result in all the opportunistically defecting co-players to contribute with probability λ if $\beta \geq c(1 - \frac{r}{N})$. The deviating individual will still, always defect. Therefore, the payoff difference after deviation considering $\beta \geq c(1 - \frac{r}{N})$ is:

   $$\pi_{\mathbf{m}}(OD,SR)-\pi_{\mathbf{m}}(OD,NR)=\lambda(N-1)(\frac{rc}{N}-\gamma) \tag{38}$$

   The deviation yields strictly positive payoff difference if $\lambda > 0$. Note that we assume always that $\gamma < \frac{rc}{N}$. So, if $\beta \geq c(1 - \frac{r}{N})$, the only value of λ for which (OC, NR) could be an equilibrium is $\lambda = 0$. At $\lambda = 0$, the strategy is exactly the same as (D, NR) and is therefore a Nash equilibrium.

   When the value of the reward is not sufficient enough for a single *SR* in the group to make the opportunists cooperate, that is: $\beta < c(1 - \frac{r}{N})$, a group of all (OD, NR) players behave exactly the same as a group of (D, NR) players. No deviation can gain a strictly higher payoff than 0 because co-players will always defect with certainty and never reward any behaviour.

5. Conditions for (OC, NR) to be an equilibrium:

   The payoff earned by a (OC, NR) player when all co-players play the same strategy is: $(1-\lambda)c(1-\frac{r}{N})$. Now let us consider a deviation from the strategy (D, NR). The payoff difference after the deviation is:

   $$\pi_{\mathbf{m}}(D,NR)-\pi_{\mathbf{m}}(OC,NR)=(1-\lambda)c(1-\frac{r}{N}) \tag{39}$$

   The payoff difference is strictly greater than 0 when $\lambda < 1$. Therefore, if (OC, NR) is a Nash equilibrium it would only be possible at $\lambda = 1$. At $\lambda = 1$, (OC, NR) is exactly the same strategy as (OD, NR) and so if $\beta < c(1 - \frac{r}{N})$ is satisfied, (OC, NR) is a Nash equilibrium. Therefore, two conditions need to hold simultaneously, $\lambda = 1$ and $\beta < c(1-\frac{r}{N})$ for (OC, NR) to be an equilibrium. □

Proof of Proposition 7. In a group composed of n_{SR} individuals playing (OC, SR) and $N - n_{SR}$ individuals playing (OC, NR), everyone will always contribute with certainty if the following holds:

$$(n_{SR} - 1)\beta \geq c(1 - \frac{r}{N}) \tag{40}$$

The above condition guarantees that all social rewarding opportunists have sufficient rewards from n_{SR}–1 co-players to cooperate. If Eq. (40) holds, then all the never rewarding opportunists will also always cooperate. The payoffs of the two strategies, under this group compostion, and the assumption on β which allows all opportunists to cooperate with certainty, are:

$$\pi_{\mathbf{m}}(OC, SR) = c(r - 1) + (n_{SR} - 1)\beta - (N - 1)\gamma$$
$$\pi_{\mathbf{m}}(OC, NR) = c(r - 1) + n_{SR}\beta$$

Now, in order to determine whether this group composition gives rise to an asymmetric Nash equilibrium, we have to check deviations for both the (OC, SR) player and the (OC, NR) player when the above condition on β holds and show that no positive difference in the payoff is gained when players deviate to a pure strategy. Let us denote the probability with which an individual, deviated to the pure strategy (σ_1, σ_2) cooperates, as p_f. Since σ_1 is a pure donor strategy, p_f can either be $0, 1, \lambda$ or $1 - \lambda$. We denote the cost of rewarding that this individual has to pay after the deviation as Γ (this depends on the contributing behaviour of the co-players of the individual and σ_2). Note that $0 \leq \Gamma \leq (N - 1)\gamma$.

First let us consider the case where the (OC, NR) individual deviates to (σ_1, σ_2). The whole group is now composed of n_{SR}, (OC, SR) players, $N - n_{SR} - 1$, (OC, NR) players and one (σ_1, σ_2) player. All the (OC, SR) and (OC, NR) co-players cooperate with certainty since the number individuals rewarding socially is sufficient for both them to cooperate opportunistically. The payoff difference of the focal individual shifting from (OC, NR) to (σ_1, σ_2) is then:

$$\pi_{\mathbf{m}}(OC, NR) - \pi_{\mathbf{m}}(\sigma_1, \sigma_2) = (1 - p_f)\left(n_{SR}\beta - c\left(1 - \frac{r}{N}\right)\right) + \Gamma \geq 0 \tag{41}$$

Thus, there is no deviation for the (OC, NR) player in this composition for which they earn a strictly higher payoff. The inequality is strict when both $\Gamma > 0$ and $p_f < 1$ and the value of reward is sufficient, Eq (40).

Now let us consider the other case when an (OC, SR) individual deviates to (σ_1, SR). As the total number of social rewarders in the group remains unchanged, everyone still cooperates with certainty. The payoff difference for the focal individual after this deviation is:

$$\pi_{\mathbf{m}}(OC, SR) - \pi_{\mathbf{m}}(\sigma_1, SR) = (1 - p_f)\left(n_{SR}\beta - c\left(1 - \frac{r}{N}\right)\right) \geq 0 \tag{42}$$

Again, considering this configuration, (OC, SR) players have no deviation to a pure strategy of the form (σ_1, SR) such that they gain a strictly higher payoff.

Finally, we consider all the deviations for the (OC, SR) player to the pure strategy of the form (σ_1, σ_2) where $\sigma_2 \in \{NR, AR, UR\}$. First let us consider the case when rewards are too valuable (opportunists cooperate even when there are $n_{SR}-2$ social rewarding co-players): $\beta(n_{SR}-1) \geq c(1-\frac{r}{N})$. It can be seen easily that a deviation to $(\sigma_1, \sigma_2) = (OC, NR)$ would result in a strictly higher payoff of $(N-1)\gamma$ to the deviating (OC, SR) individual. Therefore, the mixed configuration with n_{SR} (OC, SR) individuals and $N-n_{SR}$ (OC, NR) individuals is not a Nash equilibrium when rewards are too valuable: social rewarders have sufficient incentive to deviate to second order free riding.

Let us consider the case when rewards are not too profitable (but profitable enough): $\frac{c}{(n_{SR}-1)}(1-\frac{r}{N}) \leq \beta < \frac{c}{(n_{SR}-2)}(1-\frac{r}{N})$. That is, rewards from $n_{SR}-2$ individuals are not sufficient for opportunists to cooperate but rewards from $n_{SR}-1$ individuals are sufficient. The payoff difference of an individual deviating from (OC, SR) to (σ_1, σ_2) is:

$$\pi_{\mathbf{m}}(OC, SR) - \pi_{\mathbf{m}}(\sigma_1, \sigma_2) = (1-p_f)\left((n_{SR}-1)\beta - \left(1-\frac{r}{N}\right)c\right) + \left(\Gamma - (N-1)\gamma + \frac{\lambda(n_{SR}-1)rc}{N}\right) \quad (43)$$

This payoff difference is always greater than or equal to zero when:

$$-(N-1)\gamma + \frac{\lambda(n_{SR}-1)rc}{N} \geq 0 \implies n_{SR}-1 \geq \frac{N(N-1)\gamma}{\lambda rc} \quad (44)$$

Combining Eq (40) and (44), we get the lower bound of the number of the number of (OC, SR) in a cooperative asymmetric Nash equilibrium:

$$n_{SR}-1 \geq \max\left(\frac{c}{\beta}\left(1-\frac{r}{N}\right), \frac{N(N-1)\gamma}{\lambda rc}\right) \quad (45)$$

The upper bound for the number of (OC, SR) players in the asymmetric equilibrium comes from the condition that guarantees that it is never beneficial for the social rewarder to deviate to second order free riding when there are $n_{SR}-1$ co-players that are also rewarding socially:

$$\beta < \frac{c}{(n_{SR}-2)}(1-\frac{r}{N}) \implies (n_{SR}-1) > 1 + \left(1-\frac{r}{N}\right)\frac{c}{\beta} \quad (46)$$

□

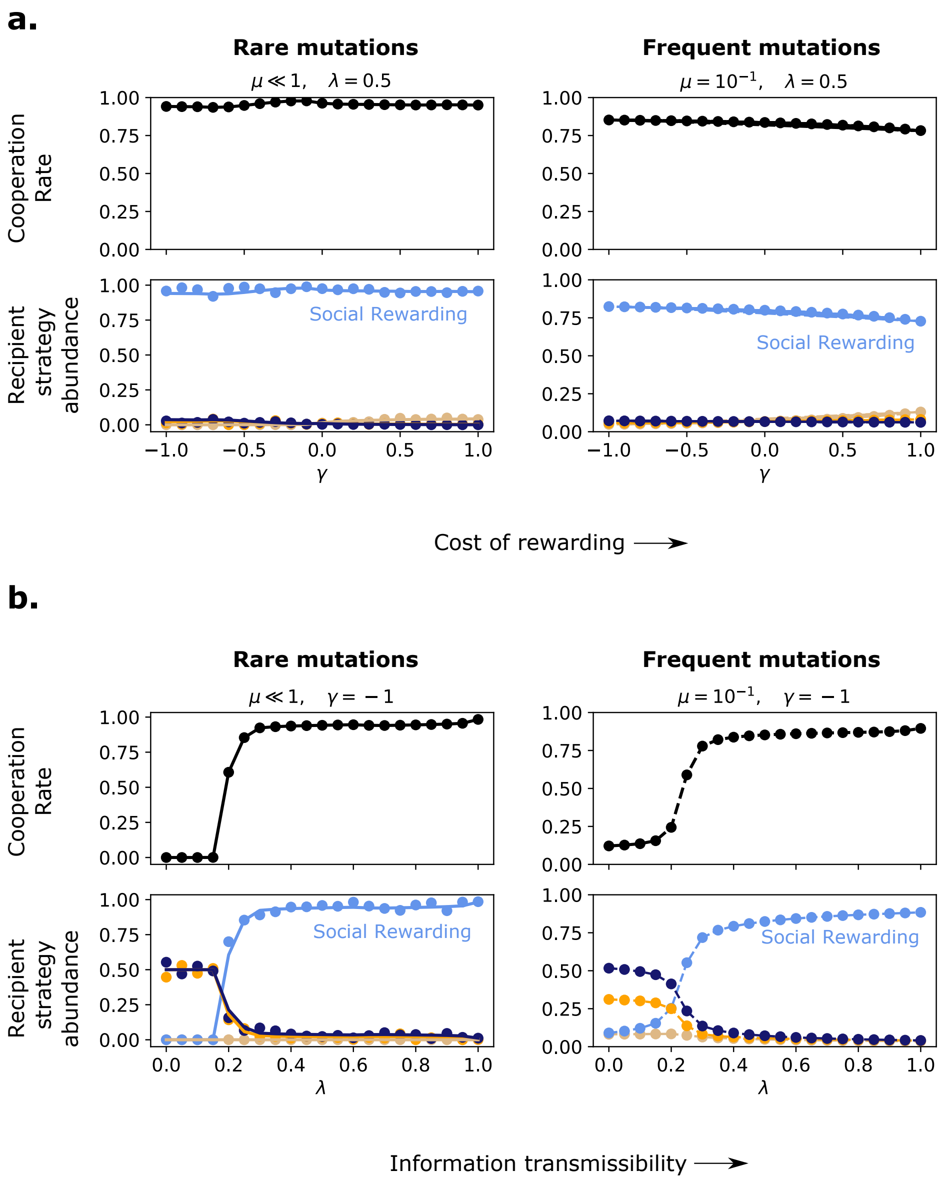

Supplementary Figure 1: Evolution of cooperation and social rewarding when rewards are mutually beneficial. In **Fig. 2**, we illustrated our evolutionary results when rewards are costly, such that $\gamma > 0$. Here we illustrate how the respective results extend to rewards that are beneficial to both parties, the donor and the recipient, by also allowing for negative values of γ. **a,** Provided that the information transmissibility of the population is sufficiently large, cooperation and social rewarding evolve even when γ is negative, irrespective of the exact mutation rate. **b,** However, as in the baseline case, cooperation and social rewarding break down for low information transmissibilities. In that case, recipients may even learn to adopt a strategy of antisocial rewarding (orange curve). Unless noted otherwise, parameters are the same as in **Fig. 2**.

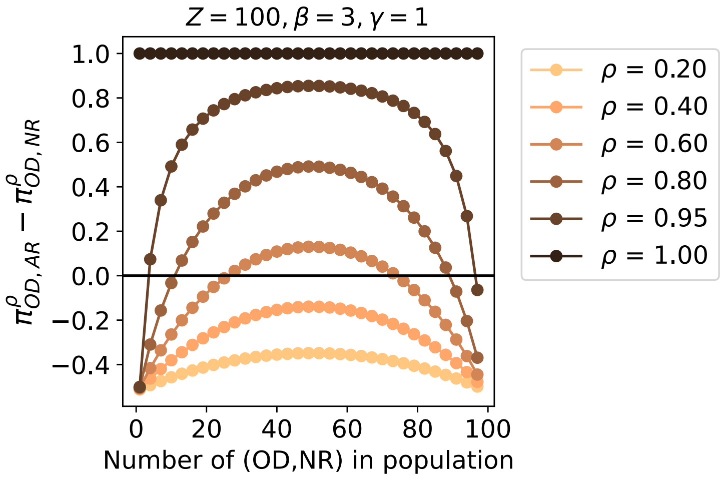

Supplementary Figure 2: Evolution of dominated strategies in assorted populations. In populations with assortment, even dominated strategies may persist. To illustrate this observation, we consider a mixed population in which i individuals adopt the strategy (*OD*,*NR*), whereas the remaining *Z*–*i* individuals adopt the strategy (*OD*,*AR*). The graph shows that although antisocial rewarding is dominated by non-rewarding, in assorted populations it may yield the larger payoff. The advantage of antisocial rewarding is particularly pronounced when assortment is strong (i.e., for large values of ρ). Plots are shown for degrees of assortment ρ ranging from 0.2 to 1.0. For the parameters we use the baseline values, $b = 4$, $\beta = 3$, $c = \gamma = 1$, in a population of size $Z = 100$. Note that for all population compositions, donors defect with certainty. Hence the value of λ does not affect the resulting payoff values.

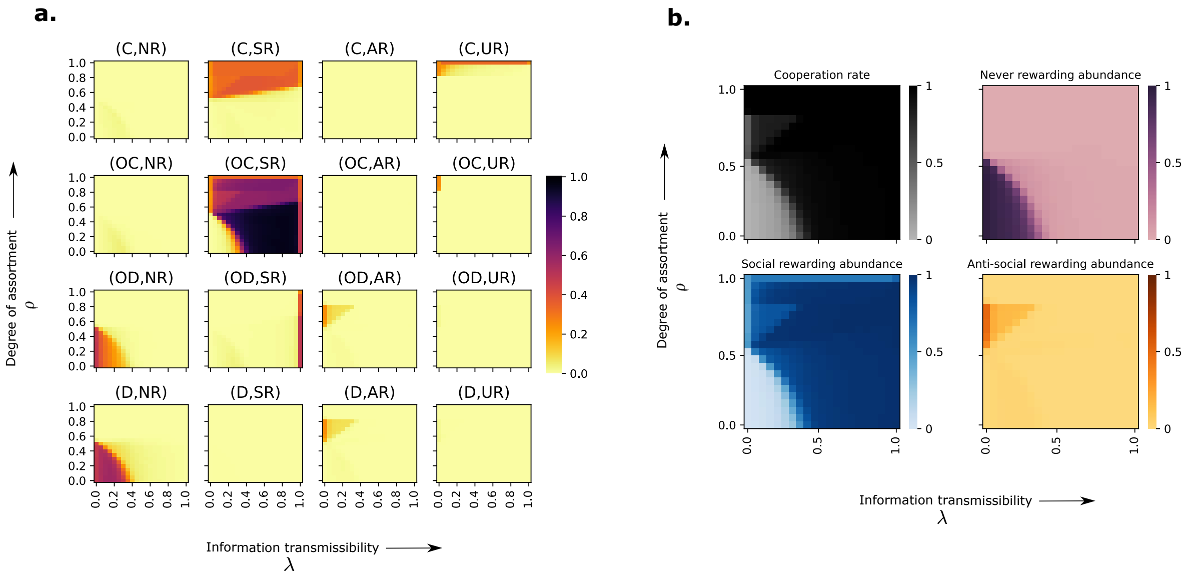

Supplementary Figure 3: Evolutionary dynamics under assortment when rewards are more profitable than cooperation. When illustrating the effect of assortment, in **Fig. 3** we assumed that the benefit of cooperation exceeds the benefit of being rewarded ($b = 4 > 3 = \beta$). Here, we repeat the respective computations for the converse case, in which rewards are more profitable than cooperation ($b = 3 < 4 = \beta$). Overall, the results are qualitatively similar. However, the parameter region in which antisocial rewarding may emerge is slightly increased. **a**, Here the relative abundances of all the 16 strategies are shown versus degree of assortment ρ and information transmissibility λ. The strategies (*OD*,*AR*) and (*D*, *AR*) emerge for a larger parameter region. **b**, The relative abundances of rewarding strategies along with average cooperation rate is shown versus degree of assortment ρ and information transmissibility λ. Except for b and β, parameter values are the same as in **Fig. 3**. Colors indicate the abundance of the respective behavior according to the stationary distribution of the process in the limit of rare mutations[3].

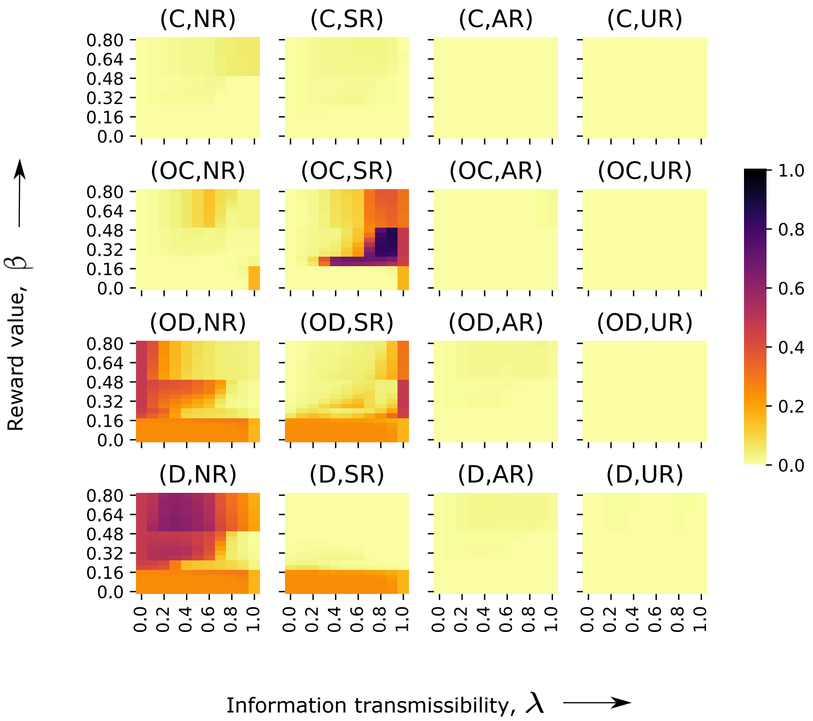

Supplementary Figure 4: Expected strategy abundances in the public good game for rare mutations. The figure complements the left panels in **Fig. 4c**, by illustrating the abundance of each strategy. As in the respective main text figure, the parameters are $c = 1$, $r = 2$, $\gamma = 0.1$, $s = 1$, $Z = 100$. The shown abundances are the numerically exact values of the pairwise comparison process in the limit of rare mutations[3].

Supplementary References

[1] Sigmund, K. *The Calculus of Selfishness* (Princeton Univ. Press, Princeton, NJ, 2010).

[2] Fudenberg, D. & Tirole, J. *Game Theory* (MIT Press, Cambridge, 1998), 6th edn.

[3] Fudenberg, D. & Imhof, L. A. Imitation processes with small mutations. *Journal of Economic Theory* **131**, 251–262 (2006).
